# Depression Levels of State Functionaries: Empirical Evidence From China

**DOI:** 10.3389/fpsyt.2021.754182

**Published:** 2021-12-13

**Authors:** Li He, Kun Wang, Zixian Zhang, Jiangyin Wang, Tianyang Li, Yuting Wang, Lixingzi Yang, Yuanyang Wu, Shuo Zhang, Siqing Zhang, Hualei Yang

**Affiliations:** ^1^School of Philosophy, Zhongnan University of Economics and Law, Wuhan, China; ^2^School of Public Administration, Zhongnan University of Economics and Law, Wuhan, China

**Keywords:** Chinese state functionary, depression, occupation, occupational health, mental health

## Abstract

This study aimed to explore the depression levels of those serving as state functionaries in China. We used data from the 2016 China Labor-force Dynamics Survey and the ordinary least squares model for the regression analysis. The results found: i) The degree of depression of state functionaries was found to be lower than that of other workers; that is, the overall depression index of state functionaries was 1.010 points lower, and the result was significant at the degree of 1%; ii) state functionaries had a lower degree of depression than workers in all other occupation groups; iii) older state functionaries had lower depression than their younger counterparts; iv) the degree of depression of state functionaries in the provinces involved in China's three major urban agglomerations was higher than that of those in other provinces; and v) the degree of depression of female state functionaries was lower than that their male peers. Thus, there is an association between serving as a state functionary in China and depression. State functionaries have lower levels of depression than other working groups. These levels were generally lower but varied according to age, sex, and province.

## Introduction

Recently, taking China's civil service exam has become increasingly popular, with more people choosing to attempt to become a state functionary. For example, the ratio of applicants to enrolment for the 2021 National Civil Service Exam was about 61 to 1 (1). This raises the question of whether serving as a state functionary provides more benefits and a happier mood. This study focused on the levels of depression experienced by state functionaries. From a theoretical perspective, this study contributes to the research on the health of workers, and especially that of civil servants. From a practical perspective, it may not only support the state in adjusting its labor and personnel policy, but also enhance social understandings of state functionaries and provide a scientific reference for people when they choose a career.

In China, state functionaries refer to persons engaged in public service in the Communist Party of China and the government and military offices at all levels of the state, public institutions, mass organizations, and state-owned enterprises. This includes persons appointed by state organs, state-owned enterprises and public institutions engaged in public service in private non-enterprise units, social organizations, and private enterprises. For example, state functionaries include the police, prosecutors, judges and other personnel in state organs performing public duties according to the law, directors, managers, accountants, and cashiers in state-owned enterprises managing and supervising state-owned property, as well as teachers and doctors in public institutions, and those who are engaged in labor service activity. However technical service jobs that do not possess authority in the aforementioned organizations, such as shop assistants and cleaners, are not commonly considered to be state functionaries. In the Chinese context, the key indicator for determining whether a person is a state functionary is “*bianzhi*”. *Bianzhi* refers to the authorized number of personnel (the number of established posts) in a party or government administrative organ, a service organization or a working unit (2), which has strong Chinese characteristics that reflect the state's functional attributes and career stability. Specifically, *bianzhi* refers to a paid job through financial appropriations and indicates that the worker cannot be dismissed. Generally speaking, only state functionaries have *bianzhi*.

Mental states can be divided into two types: mental health and mental problems. Mental problems consist of three levels: poor states, mental disorders, and mental illness. The World Health Organization pointed out that mental problems can significantly impact all areas of life, and the two most common mental disorders are depression and anxiety (3). As far as the relationship between state functionaries and depression is concerned, some conflicting conclusions exist in the existing literature. Most scholars have observed that state functionaries tend to have higher levels of depression. For example, in terms of doctors as state functionaries, Wall et al. (4) found, in a study of 11,000 British health professionals, that they had a higher prevalence of minor psychiatric disorders than in the general population. Since then, scholars in other countries have found a particularly high prevalence of depression among doctors (5–10). In terms of teachers as state functionaries, Jurado et al. (11) found that working in public schools increased the risk of depression for teachers after studying teachers in public and private schools. In summary, not only do teachers have high levels of depression, but their average health scores are also lower than those reported in the sample of the working population (12). These conditions also exist in China (13). In terms of other occupations as state functionaries, Stansfeld et al. (14) found that people working as machine operators and in the handicraft industry, as well as those in professional and technical occupations have a lower prevalence of common mental disorders, while government officials and administrators have a higher prevalence.

In general, prison officers, police, ambulance personnel, etc. scored below average in physical health, mental health, job satisfaction, and other aspects (15). All of these studies support the idea that state functionaries have higher levels of depression.

However, some scholars have arrived at different conclusions. For example, in a related study on government employees, Nahar et al. (16) found that government employees had lower levels of depression and better mental health by comparing government employees with non-government employees in Bangladesh. In the relevant research on employees of public sector banks, Jogsan (17) studied bank employees and found that compared with employees of public sector banks, employees of private sector banks had worse mental health and higher levels of depression. Although there is a general consensus among scholars around the world that there is a link between state functionaries and depression, some have found no such link for specific occupations of state functionaries. For example, there is no evidence that doctors, teachers, etc., have higher levels of depression than other occupational groups (18–20).

Significant research on the relationship between other occupational groups and depression has been conducted. Grosch and Murphy (21) found that the prevalence of depression was lower among professionals and managers and higher in occupations involving operating machinery or transporting equipment. Subsequently, Sanne et al. (22) carried out a more detailed analysis on whether and how the degree of anxiety and depression varies between different occupations. They found that occupational skill level negatively correlated with the prevalence of anxiety and depression. In other words, people working in primary occupations, such as agriculture and forestry, the handicraft industry, machine operators, and assemblers, have a higher prevalence of anxiety and depression than professionals, technicians, and professional assistants. Scholars in different countries have also carried out similar studies on the working population to verify whether the conclusions were applicable. Separate studies on working populations in France, the United States, and Norway arrived at similar conclusions. That is, the higher the skill level of the occupation, the lower the prevalence of depression (23–26). Additionally, although studies from various countries have demonstrated that there is a close relationship between other occupational groups and depression, some scholars have argued that there is no apparent relationship between these two factors (27, 28).

Compared with previous research, this study focused on depression levels among state functionaries compared to other occupations. We used data from the 2016 China Labor-force Dynamics Survey (CLDS), working workers since 2015 as the research object, and the ordinary least squares (OLS) model for the regression analysis. The sequence of our study was as follows. First, the levels of depression experienced by state functionaries in China was investigated; second, a robustness test was carried out by changing the explained variable and using propensity score matching (PSM) to verify the reliability of the results; third, a heterogeneity analysis was carried out using age, region, and sex to explore the differences in depression levels among state functionaries; and finally, we compared the degree of depression of different types of occupation with that of state functionaries.

## Materials and Methods

### Data

The data used in this study were obtained from the 2016 CLDS, conducted by Sun Yat-sen University. The survey takes the labor force as the survey object. It explores issues such as labor education, employment, labor rights, occupational mobility, occupational protection, health, and occupational satisfaction. The 2016 survey was a large-scale interdisciplinary follow-up survey. It used a multi-stage, multi-level, probability sampling method proportional to the size of the labor force to determine the survey sample, which covered 29 provinces and cities in China, involving 21,086 people. Considering the applicability of the problem, the present study restricted the research object to members of the labor force who have had work experience since January 2015. After we had excluded missing values and illogical samples, the effective analysis sample size was 14,147.

### Variables

#### Depression

Depression levels in this study were obtained by calculating the scores of the Center for Epidemiologic Studies Depression (CES-D) Scale. The CES-D scale was compiled by Radloff (29) and is widely used in mental health research (30–32).

#### State Functionary

In the Chinese context, the key indicator for determining whether a person is a state functionary is “*bianzhi*”. *Bianzhi* refers to the authorized number of personnel (the number of established posts) in a Party or government administrative organ, a service organization or a working unit (2), which has strong Chinese characteristics that reflect the state's functional attributes and career stability. Specifically, *bianzhi* refers to a paid job through financial appropriations and indicates that the worker cannot be dismissed. Generally speaking, only state functionaries have *bianzhi*. Therefore, this study defined those with *bianzhi* as state functionaries and assigned these a value of 1; those without *bianzhi* were non-state functionaries and were given a value of 0.

#### Control Variables

Based on the research aims, focusing on the depression of workers and referring to the research of Marchand et al. (33), this study considered several control variables, including sex, age, health, religious beliefs, sense of justice, marital status, social interaction, smoking, alcohol use, exercise, and province.

### Model Selection

Because the depression score is a continuous variable and the missing variables and measurement errors are well controlled, this study selected the OLS model for the regression analysis. The model was set as follows.


(1) $$\begin{array}{l} depress_{i}=\alpha_{0}+\alpha_{1}occupation_{i}+\alpha_{2}X_{i}+\varepsilon_{i} \end{array}$$

where *depress*_*i*_ denotes the degree of depression of the i-th respondent; *occupation*_*i*_ denotes whether the i-th respondent is a state functionary; *X*_*i*_ denotes other control variables; ε_*i*_ denotes the random error term; and α_1_ is the coefficient to be estimated in this study, reflecting the influence of serving as a state functionary on depression.

To deal with the endogeneity problem caused by self-selection, this study adopted the PSM method proposed by Rosenbaum and Rubin (34) to match the sample of individuals serving as state functionaries to control the self-selection effect of state functionaries to a greater extent and reduce the selectivity bias and endogenous problems (35). In this study, individuals who served as state functionaries were considered the treatment group, and individuals who were not state functionaries as the control group. The specific steps were as follows: first, we calculated the conditional probability of an individual serving as a state functionary, that is, the propensity score (PS).


(2) $$\begin{array}{l} \text{PS}(\text{X})=\text{Pr}\left\{\text{D}=1|\text{X}\right\}=\text{E}\left\{\text{D}|\text{X}\right\} \end{array}$$

where D is a dummy variable for whether the individual is a state functionary. If the individual was a state functionary, then D = 1, otherwise D = 0. X is all the covariates that determine whether an individual is a state functionary, which means that given X, the depression level of an individual has negligible influence on whether the person is a state functionary. Drawing lessons from the research ideas of Rajeev and Sadek (36), this study selected the Logit model to estimate:


(3) $$\begin{array}{l} \text{PS}(\text{Xi})=\text{Pr}\;\left\{\text{Di}=1|\text{Xi}\right\}=\text{exp}\;\left(\beta \text{Xi}\right)/\left(1+\text{exp}\;\left(\beta \text{Xi}\right)\right) \end{array}$$

where β is the regression coefficient of the Logit model, and PS is the predicted value of the Logit model.

Second, the treatment group was matched to the control group according to the PS value. This study utilized the currently commonly used nearest neighbor matching method: that is, according to the PS value, the treatment group and the control group are searched to determine the group with the smallest absolute difference in PS and the nearest neighbor for pairing. After matching, the control group retained only the individuals with the closest individual characteristics to the treatment group and excluded individuals with large differences in individual characteristics from the treatment group.

Finally, this study conducted a balance test to determine the effectiveness of the matching effect between the treatment group and the control group.

## Results

### Descriptive Statistics

Table 1 reports the descriptive statistical results of the sample. The average depression of the sample is 27.039, and individuals who work as state functionaries account for 7.4% of the total. In terms of other characteristics of the sample, males accounted for 53.6%, and the average age was 45.7 years. People with religious beliefs accounted for 12.7%; married people accounted for 86.9%; smokers accounted for 32.3%, and alcoholics for 23.4%; about 28.4% of individuals regularly engage in exercise.

**Table 1:** Variable definitions and descriptive statistics.

**Variable code**	**Variable definition**	**Obs**	**Mean**	**Std dev**.	**Min**	**Max**
Depression	20 negative emotion questions, each question has 4 options: no = 1 point, rarely = 2 points, often = 3 points, almost always = 4 points. Add up the scores of the 20 questions, the higher the score, the more depressed.	14,147	27.039	8.68	20	80
Occupation	State functionary = 1, otherwise = 0	14,147	0.074	0.261	0	1
Sex	Male = 1, female = 0	14,147	0.536	0.499	0	1
Age	The actual age of the respondent	14,147	45.714	13.002	15	96
Health	Self-rated health, very healthy = 1, healthy = 2, general = 3, relatively unhealthy = 4, very unhealthy = 5	14,147	2.355	0.966	1	5
Religious beliefs	No religious belief = 0, have religious belief = 1	14,147	0.127	0.333	0	1
Sense of justice	Completely unfair = 1, relatively unfair = 2, not fair but also not unfair = 3, relatively fair = 4, completely fair = 5	14,147	3.286	0.936	1	5
Marital status	First marriage, remarriage = 1, unmarried, divorced, widowed, cohabitation = 0	14,147	0.869	0.338	0	1
Social interaction	Very unfamiliar = 1, not very familiar = 2, general = 3, relatively familiar = 4, very familiar = 5	14,147	3.814	0.99	1	5
Smoke	Smoking = 1, no smoking = 0	14,147	0.323	0.468	0	1
Drink	Using alcohol = 1, not using alcohol = 0	14,147	0.234	0.424	0	1
Exercise	Exercise = 1, no exercise = 0	14,147	0.284	0.451	0	1
Province	Provinces involved in the three major urban agglomerations = 1, others = 0	14,147	0.302	0.459	0	1

### Benchmark Regression

Model 1 considered only the main explanatory variable of occupation. Model 2 added the individual demographic variables of sex, age, and health, based on Model 1. Model 3 added the individual psychological variables of religious beliefs and sense of justice based on Model 2. Model 4 added the social relationship variables of marital status and social interaction based on Model 3. Model 5 added the health variables of smoking, alcohol use, and exercise based on Model 4 and controlled for the province variable. The interpretation of the results was based on Model 5. It can be seen from Model 5 that serving as a state functionary is linked to a reduction in depression. Compared with non-state functionaries, the mental health of state functionaries is better, and their depression score is 1.010 points lower (see Table 2).

**Table 2:** Benchmark regression results.

	**Model 1**	**Model 2**	**Model 3**	**Model 4**	**Model 5**
Occupation	−1.822^***^	−1.016^***^	−0.884^***^	−1.038^***^	−1.010^***^
	(0.239)	(0.236)	(0.234)	(0.236)	(0.240)
Sex		−1.482^***^	−1.437^***^	−1.451^***^	−1.591^***^
		(0.140)	(0.138)	(0.139)	(0.176)
Age		−0.014^**^	−0.005	0.013^**^	0.008
		(0.006)	(0.006)	(0.006)	(0.006)
Health		2.787^***^	2.552^***^	2.505^***^	2.480^***^
		(0.087)	(0.086)	(0.087)	(0.087)
Religious beliefs			0.979^***^	1.036^***^	0.983^***^
			(0.219)	(0.218)	(0.217)
Sense of justice			−1.506^***^	−1.495^***^	−1.520^***^
			(0.082)	(0.082)	(0.082)
Marital status				−0.966^***^	−1.124^***^
				(0.216)	(0.217)
Social interaction				−0.472^***^	−0.550^***^
				(0.076)	(0.076)
Smoke					0.223
					(0.177)
Drink					0.069
					(0.169)
Exercise					−0.512^***^
					(0.147)
Province					−1.603^***^
					(0.141)
_cons	27.173^***^	21.976^***^	26.936^***^	28.833^***^	30.266^***^
	(0.077)	(0.282)	(0.399)	(0.470)	(0.493)
N	14,147	14,147	14,147	14,147	14,147
R-square	0.003	0.106	0.133	0.137	0.145

* *p < 0.1*,

** *p < 0.05*,

*** *p < 0.01. Robust standard errors are reported*.

In terms of the control variables, female depression was significantly slighter than male depression. The better the physical health, the lower the corresponding depression. Religious people had significantly higher depression scores than non-religious people, while individuals with a weaker sense of justice had more serious depression. Compared with unmarried people, married people had lower levels of depression. People with higher levels of social interaction and people who exercise more also had less depression. Compared with the residents of the provinces involved in the three major urban agglomerations (Beijing and surrounding cities, Shanghai and surrounding cities, and Guangzhou and surrounding cities; the development level of Hebei Province is lower than Beijing and Tianjin, so it was excluded from the sample), residents from other provinces had lower levels of depression.

Age, smoking, and alcohol use had no significant impact on the workers' depression levels. However, the older people were, the higher they scored for depression. At the same time, people who smoked and used alcohol had more serious depression.

### Robustness Test

To ensure the reliability of the conclusions, this study further examined the influence of serving as a state functionary on depression by replacing the explained variable and the PSM method. The results are illustrated in Table 3. Model 6 used life satisfaction to measure depression. Unlike the depression level, life satisfaction is a positive indicator. The answers to life satisfaction in the CLDS are divided into five levels: “very dissatisfied,” “relatively dissatisfied,” “average,” “relatively satisfied,” and “very satisfied.” In this study, the values were 1, 2, 3, 4, and 5, respectively; the higher the life satisfaction score, the lower the depression. To solve the problem of the self-selection of the samples, the 1:1 nearest neighbor matching method was used to screen out workers with similar basic conditions as state functionaries so that state functionary became the result of random allocation. Table 4 illustrates the results of the balance test. The deviation of all characteristic variables after matching was <10%, which proved that the balance test was passed. Model 7 is the result of the regression based on the matching samples. Judging from the results in Table 4, regardless of the method adopted, the significance and signs of the impact of serving as a state functionary on depression levels did not change. Therefore, the regression results of this study were generally robust.

**Table 3:** Robustness test.

**Variable code**	**Model 6**	**Model 7**
	**Happiness**	**Propensity score**
Occupation	0.572^***^	−0.612^*^
	0.061	0.317
Sex	−0.118^***^	−1.104^***^
	0.040	0.400
Age	−0.003^**^	−0.013
	0.001	0.017
Health	−0.455^***^	1.596^***^
	0.020	0.198
Religious beliefs	0.153^***^	−0.484
	0.047	0.664
Sense of justice	0.534^***^	−1.202^***^
	0.020	0.179
Marital status	0.381^***^	−0.605
	0.052	0.524
Social interaction	0.188^***^	−0.518^***^
	0.018	0.162
Smoke	0.001	0.346
	0.043	0.422
Drink	0.062	0.268
	0.042	0.413
Exercise	0.407^***^	−0.398
	0.037	0.317
Province	−0.068^*^	−0.971^***^
	0.036	0.360
_cons	0.204	30.489^***^
	0.129	1.111
N	14,147	2,188
R-square	0.065	0.076

* *p < 0.1*,

** *p < 0.05*,

*** *p < 0.01. Robust standard errors are reported*.

**Table 4:** Balance test.

**Variable code**	**Unmatched**	**Mean**		**Bias%**	**Reduct bias%**	***T*-value**	***P*-valve**
	**Matched**	**Treated**	**Control**				
Sex	U	0.619	0.542	15.7		4.81	0.000
	M	0.619	0.614	1.0	93.8	0.23	0.821
Age	U	42.644	45.829	−27.9		−7.72	0.000
	M	42.656	41.888	6.7	75.9	1.66	0.096
Health	U	2.112	2.355	−27.3		−7.90	0.000
	M	2.113	2.039	8.3	69.5	2.01	0.044
Religious beliefs	U	0.0684	0.130	−20.7		−5.76	0.000
	M	0.068	0.061	2.3	89.0	0.62	0.533
Sense of justice	U	3.354	3.283	7.6		2.34	0.019
	M	3.353	3.343	1.0	86.4	0.24	0.812
Marital status	U	0.865	0.870	−1.6		−0.49	0.625
	M	0.865	0.879	−4.3	−172.1	−0.99	0.324
Social interaction	U	3.417	3.847	−42.9		−13.51	0.000
	M	3.420	3.431	−1.2	97.3	−0.25	0.799
Smoke	U	0.334	0.331	0.6		0.20	0.841
	M	0.335	0.343	−1.8	−184.7	−0.42	0.676
Drink	U	0.278	0.237	9.4		2.98	0.003
	M	0.278	0.265	2.9	69.5	0.64	0.521
Exercise	U	0.547	0.267	59.6		19.44	0.000
	M	0.547	0.527	4.1	93.1	0.88	0.379
Province	U	0.265	0.303	−8.5		−2.60	0.009
	M	0.265	0.275	−2.1	74.9	−0.49	0.621

### Heterogeneity Analysis

Does age, region, and sex affect the relationship between being a state functionary and depression? On the basis of this question, we examined the level of depression and the effect of being a state functionary on depression levels by age, region, and sex grouping. The results are presented in Tables 5, 6, respectively. Table 5 illustrates that the mean and median depression levels are higher among older age groups. In terms of region, the mean and median depression levels were higher in the provinces not involved in the three major urban agglomerations. In terms of sex, the depression mean and median were higher for females.

**Table 5:** Subgroup depression status.

**Depression**	**Age**		**Region**		**Sex**	
	**Young (age < = 45)**	**Old (age >45)**	**Provinces involved in the three major urban agglomerations**	**Other provinces**	**Male**	**Female**
Mean	26.441	27.567	26.024	27.479	26.179	28.031
Median	23	24	23	24	23	25
N	6633	7514	4273	9874	7578	6569

**Table 6:** Heterogeneity analysis.

**Variable code**	**Model 9**		**Model 10**		**Model 11**	
	**Young (age < = 45)**	**Old (age > 45)**	**Provinces involved in the three major urban agglomerations**	**Other provinces**	**Male**	**Female**
Occupation	−0.822^***^	−0.984^***^	−0.156	−1.271^***^	−0.753^**^	−1.471^***^
	0.314	0.381	0.397	0.295	0.314	0.364
Sex	−0.994^***^	−2.220^***^	−1.416^***^	−1.667^***^		
	0.233	0.265	0.299	0.216		
Age	−0.014	0.016	−0.028^***^	0.023^***^	−0.007	0.022^**^
	0.015	0.014	0.010	0.008	0.008	0.010
Health	2.120^***^	2.669^***^	2.543^***^	2.437^***^	2.094^***^	2.869^***^
	0.133	0.114	0.155	0.104	0.113	0.132
Marital status	−0.825^***^	−0.899^**^	−1.099^***^	−0.974^***^	−0.750^***^	−1.379^***^
	0.276	0.432	0.331	0.282	0.263	0.362
Religious beliefs	0.978^***^	0.976^***^	1.299^***^	0.917^***^	0.434	1.413^***^
	0.281	0.337	0.364	0.268	0.272	0.333
Sense of justice	−1.333^***^	−1.706^***^	−1.567^***^	−1.520^***^	−1.378^***^	−1.662^***^
	0.109	0.121	0.129	0.103	0.103	0.129
Social interaction	−0.534^***^	−0.554^***^	−0.414^***^	−0.593^***^	−0.529^***^	−0.628^***^
	0.096	0.119	0.109	0.099	0.100	0.116
Smoke	0.175	0.375	−0.078	0.363^*^	0.195	1.701^**^
	0.256	0.246	0.297	0.219	0.180	0.828
Drink	0.167	0.058	0.539^*^	−0.112	−0.093	0.942^*^
	0.249	0.229	0.284	0.208	0.178	0.509
Exercise	−0.625^***^	−0.458^**^	−0.611^***^	−0.528^***^	−0.472^**^	−0.576^**^
	0.195	0.220	0.230	0.188	0.190	0.228
Province	−1.089^***^	−2.090^***^			−1.574^***^	−1.649^***^
	0.194	0.207			0.182	0.219
_cons	30.349^***^	30.185^***^	29.569^***^	29.774^***^	29.476^***^	29.581^***^
	0.702	1.101	0.757	0.624	0.629	0.780
N	6633	7514	4273	9874	7578	6569
R-square	0.106	0.170	0.162	0.135	0.115	0.160

* *p < 0.1*,

** *p < 0.05*,

*** *p <0.01. Robust standard errors are reported*.

The results of the heterogeneity analysis are specified in Table 6. Model 9 illustrates that both older and younger people serving as state functionaries had significantly slighter depression. Compared with young people, the effect of serving as a state functionary on the depression levels of older people was stronger. Model 10 demonstrates that serving as a state functionary in the provinces not involved in the three major urban agglomerations significantly affected reducing depression while serving as a state functionary in the provinces involved in the three major urban agglomerations had a positive but not significant impact. From the sex difference perspective in Model 11, both male and female state functionaries had better mental health, and the degree of depression of the latter was lower than that of the former.

### Further Analysis

To study the depression differences between state functionaries and people working in other specific occupation types, we conducted further analyses of the occupational sub-samples. The CLDS questionnaire has eleven responses to occupation type: “Party and government agencies, people's organizations, military,” “State-owned/collective public institutions,” “State-owned enterprises,” “Collective enterprises,” “Village neighborhood committees and other autonomous organizations,” “Private enterprises,” “Foreign companies,” “Social organizations,” “Self-employed workers,” “Peasants,” and “Freelance workers.” State functionaries are distributed in the first four types. Therefore, workers in the first four types with no *bianzhi* were combined into a non-*bianzhi* group. Table 7 reports the results of depression levels of state functionaries compared to practitioners in other occupational types. Among them, state functionaries were assigned a value of 1, and all other types of employees being compared were assigned a value of 0. Model 12 shows the results of the full sample regression. From model 13 to model 20, the results of the comparison of depression levels between state functionaries and non-bianzhi workers, practitioners in autonomous organizations, private enterprises, foreign companies, and social organizations, self-employed workers, peasants, and freelance workers, are shown respectively. From all the models in Table 7, it can be seen that state functionaries had a lower level of depression than those in any other type of occupation.

**Table 7:** Further analysis.

	**Model 12**	**Model 13**	**Model 14**	**Model 15**	**Model 16**	**Model 17**	**Model 18**	**Model 19**	**Model 20**
	**Full sample**	**Non-bianzhi**	**Autonomous organization**	**Private enterprise**	**Foreign companies**	**Social organization**	**Self-employed work-ers**	**Peas-ants**	**Free-lancer**
Occupation	−1.010^***^	−0.752^**^	−1.611^***^	−0.545	−0.811	−0.884	−0.908^*^	−1.418^***^	−0.583
	0.240	0.361	0.592	0.392	0.597	0.616	0.510	0.320	0.480
Sex	−1.591^***^	−0.851^*^	−2.453^***^	−1.771^***^	−2.326^***^	−2.190^***^	−1.999^***^	−1.885^***^	−1.324^***^
	0.176	0.451	0.545	0.324	0.512	0.551	0.405	0.240	0.423
Age	0.008	−0.010	0.011	0.008	−0.016	−0.010	0.026	−0.013	0.029^*^
	0.006	0.020	0.023	0.012	0.020	0.022	0.017	0.008	0.016
Health	2.480^***^	1.490^***^	2.553^***^	2.371^***^	2.447^***^	2.446^***^	1.816^***^	2.775^***^	2.424^***^
	0.087	0.257	0.279	0.164	0.275	0.286	0.206	0.117	0.204
Religious beliefs	0.983^***^	1.653^*^	1.075	0.257	0.733	0.833	0.709	2.160^***^	0.627
	0.217	0.864	0.957	0.346	0.761	0.862	0.684	0.429	0.408
Sense of justice	−1.520^***^	−1.123^***^	−1.825^***^	−1.740^***^	−1.869^***^	−1.918^***^	−1.956^***^	−1.488^***^	−1.347^***^
	0.082	0.206	0.252	0.154	0.249	0.260	0.202	0.107	0.201
Marital status	−1.124^***^	−0.308	−0.213	−1.053^***^	−0.046	0.027	−1.236^**^	−0.972^***^	−0.588
	0.217	0.553	0.678	0.407	0.597	0.657	0.587	0.290	0.544
Social interaction	−0.550^***^	−0.683^***^	−0.527^**^	−0.519^***^	−0.681^***^	−0.475^*^	−0.633^***^	−0.360^***^	−0.517^***^
	0.076	0.177	0.253	0.139	0.226	0.248	0.217	0.098	0.186
Smoke	0.223	0.373	1.242^**^	0.632^**^	1.373^**^	1.330^**^	0.853^**^	0.385	0.308
	0.177	0.494	0.561	0.321	0.532	0.573	0.427	0.237	0.463
Drink	0.069	0.570	1.309^**^	−0.138	0.731	1.033^*^	0.959^**^	0.384^*^	0.077
	0.169	0.484	0.567	0.298	0.517	0.609	0.420	0.229	0.457
Exercise	−0.512^***^	−0.935^***^	−0.391	−0.885^***^	−0.846^*^	−0.810^*^	−0.549	−0.323	−0.873^**^
	0.147	0.357	0.468	0.264	0.447	0.473	0.345	0.204	0.370
Province	−1.603^***^	−0.808^**^	−1.144^**^	−1.236^***^	−0.955^*^	−0.944^*^	−1.034^**^	−1.560^***^	−1.621^***^
	0.141	0.369	0.540	0.258	0.532	0.542	0.503	0.188	0.510
_cons	30.266^***^	30.602^***^	29.953^***^	31.012^***^	31.757^***^	30.720^***^	32.301^***^	29.447^***^	28.713^***^
	0.493	1.306	1.649	0.923	1.551	1.691	1.264	0.673	1.213
N	14147	1832	1312	3805	1292	1104	2460	7276	2360
R-square	0.145	0.074	0.176	0.154	0.192	0.185	0.131	0.171	0.126

* *p < 0.1*,

** *p < 0.05*,

*** *p < 0.01. Robust standard errors are reported*.

## Discussion

### The Degree of Depression of State Functionaries Is Lower

In our comparison of the degree of depression of state functionaries and that of others, we focused on the differences in the characteristics of their occupations. We focused on the differences in income and working hours and the influence of personal factors.

An increase in personal income will give workers a stronger sense of security and happiness, while income stability can have similar effects. Compared with non-state functionaries, state functionaries have more stable jobs and incomes, bear less unemployment risk and face less internal competition (16). This provides state functionaries with a stronger sense of security, happiness, and confidence in life.

Work-life balance also plays a role. Long work hours can lead to a deterioration of people's psychological state (37). The working hours and rest time of most state functionaries are relatively fixed, and their rest time is also sufficient. The rest time helps to ease their psychological pressure, making them less prone to depression.

There is a positive correlation between education degree and mental health awareness (38, 39). Compared with non-state functionaries, the general education degree of state functionaries is higher, which helps them pay more attention to psychological problems. When a problem arises, such as depression, they can often identify it earlier and seek help from professionals.

In other words, lower life pressure, more sufficient rest time, and a stronger mental health consciousness mean that the degree of depression of state functionaries is lower than that of non-state functionaries.

### The Degree of Depression of Older State Functionaries Is Lower

Life experience is the most conspicuous difference between the older and younger state functionaries. Life experience brings about differences in matters such as authority, living conditions, and work experience.

The promotion of state functionaries is also implicated in the question of seniority. Older people tend to have been promoted to higher positions of authority, and they do not have to work as hard as younger workers. Younger people, on the contrary, are in a lower position and must deal with more complicated affairs due to the pressure of job responsibilities. This extra work causes a greater psychological burden (40). Additionally, young people are also under pressure to get promoted. To pursue higher positions, they need to achieve higher work goals. These demands on work performance cause long-term pressure (41), which can easily lead to mental health problems, especially depression.

Generally speaking, senior state functionaries have higher income, more savings, and relatively stable family expenditure. Conversely, young state functionaries have low incomes and face the challenges of setting up a home, getting married, raising children, and supporting them. In other words, the older state functionaries have gradually entered a mature and stable stage in terms of family, career, and other aspects, with better living conditions (42). Higher income levels and better living conditions often mean a greater risk resilience. In general, the older state functionaries have better coping abilities than the younger ones.

With more experience, older people are better able to cope with complex and unexpected situations at work. First, older state functionaries have a long service life, have experienced more difficult situations in their work, and have more experience in dealing with challenges. Second, rich work experience enables older people to have a better attitude than younger people, especially when facing emergencies (43). This can significantly reduce their psychological stress in the face of difficulties. Young people are more likely to be fearful when faced with difficulties they have never experienced (44).

Therefore, the impact of age factors on the degree of depression of state functionaries mainly concerns family and work. Work–family conflict can affect an individual's mental health, including depression (45, 46). Young state functionaries are just entering their posts and may have both family and work pressure as they adjust to their jobs. Older people not only have the experience of dealing with family and work problems but can also find a better balance between family and work to avoid possible conflicts between the two (47). These factors contribute to the lower degree of depression in older state functionaries than in younger ones.

### The Degree of Depression of State Functionaries in Provinces Outside the Three Major Urban Agglomerations Is Lower

The difference between the provinces involved and provinces not involved in the three major urban agglomerations lies in the degree of social and economic development. Comparatively speaking, the degree of economic development of the provinces involved in the three major urban agglomerations is higher than the other provinces, but this also creates higher living costs and life and work expectations. These can easily lead to depression and anxiety (48).

Relative income is low because, compared to many other occupations, the income of Chinese state functionaries is not high. Additionally, there is less difference in the incomes of state functionaries in similar positions from different regions. This means that state functionaries from economically developed provinces face higher living costs than other provinces and have a wider income gap than other occupational groups. Excessive financial stress caused by the cost of living and the income gap can lead to depression (49).

High demands result in high pressure (50). The economic and social development of the provinces involved in the three major urban agglomerations is mature, public affairs are numerous, and government affairs are more complicated. To cope with the above situation, the relevant governments continue to promote reform and innovation, improve competition, evaluation, and other mechanisms, and require more from state functionaries. On the contrary, other provinces are lagging in the above aspects of reform. High demands cause high stress, and state functionaries who have been under high stress for a long time are more likely to suffer from depression.

In conclusion, the contradiction between income and living costs and the higher work requirements mean that the state functionaries involved in the three major urban agglomerations bear more psychological pressure than those who are not in those provinces.

### The Degree of Depression of Female State Functionaries Is Lower

When researching depression degrees of state functionaries, we must consider the impact of gender differences. The physical and psychological differences between women and men are closely related to their performance in work and life.

There are differences in the degree of family connection between men and women. Most state functionaries usually consider starting a family when they have a more stable income. Female state functionaries have a higher degree of family connection. Most women value their families more than men and are willing to spend more time on family life (51). Women are also better at turning to family members for help in times of psychological stress, which can provide an effective buffer against stress (52).

Family and society have different expectations of men and women. Traditional beliefs in many contexts hold that women have a lower societal position (53) and that family and society have lower career expectations for women than for men. In China, men are not only expected to bear the lion's share of their family's cost of living but also a part of the burden of the previous generation. Conversely, women are less likely to be exposed to similar pressures. The particular position of state functionaries determines that their income will not be too high. In this case, male state functionaries bear more life pressure than female state functionaries.

Additionally, there is a gender gap in promotion opportunities. In the global world of work, the proportion of female leaders is much smaller than that of male leaders (54, 55), and China is no exception. This is partly a sign that women have fewer chances of promotion than men. It is difficult for women to get the same recognition, even if they put in equal or even greater effort than men. The mismatch between effort and gain has led many female state functionaries to abandon the competition for promotions. When they lose their hopes of promotion, female state functionaries often no longer pursue outstanding performance at work and become less sensitive to job gains and losses, reducing their psychological pressure and risk of depression.

Based on the above analysis, we can conclude that most female state functionaries have less pressure and are less likely to suffer from depression than men. However, this conclusion is based on a specific group of state functionaries. From a general societal perspective, women are often discriminated against at work due to the shackles of traditional concepts. They are more family-oriented, making them more vulnerable when faced with work–family conflicts (56). Therefore, the mental health status of this female group will be worse (57), and they are more likely to have depression.

### The Degree of Depression in Private Enterprises and Foreign Companies, Peasants and Self-Employed Workers Is Higher

State functionaries have lower levels of depression than those in private enterprises and foreign companies, but the advantage is not significant. Due to different management characteristics, private enterprises and foreign companies are considered to be unstable. Heavier tasks, longer working hours, and relatively poor working environments can have a negative impact on employees' mental health. Staff in these environments operate under greater work and psychological pressure, and depression levels are higher than state functionaries (58, 59). However, in terms of depression levels, the difference is small. People in private enterprises and foreign companies have higher incomes. Thus, the effect of income can compensate for the negative effects of work environments on mental health, and there is less difference in depression levels compared with state functionaries. The welfare of employees of private enterprises and foreign companies are guaranteed to some extent. This can effectively relieve their mental stress and reduce their depression levels. Therefore, although the depression levels of private enterprises and foreign companies are relatively higher than that of state functionaries, the difference is not significant.

State functionaries also have lower levels of depression than peasants and self-employed workers, and the advantage is significant. Peasants and self-employed workers are more vulnerable than workers in private enterprises and foreign companies. Vulnerability is the inability of individuals or social groups to cope with or adapt to disaster-induced stress placed on their livelihood and well-being (60). On the one hand, although most peasants and self-employed workers can relieve themselves of work pressure through free rest time, their work and income are not guaranteed by the labor system and minimum wage requirements. On the other hand, the life and work of peasants and self-employed workers are highly uncertain and easily affected by changes in the natural and social environment. This vulnerability makes them bear greater psychological pressure, and their depression levels rise easily when affected by negative factors from outside. In addition, China has a strong “official rank standard” culture and the social recognition of peasants and self-employed workers is low. This makes them more susceptible to occupational discrimination in their work and life, while perceived discrimination may have a negative impact on their mental health (61), which is also related to the generation of depression (62). Therefore, compared with state functionaries, peasants and self-employed workers have higher levels of depression, and the difference is significant.

### The Influence of Control Variables on the Degree of Depression

The benchmark regression results suggested that people with religious beliefs had higher levels of depression in China, which was also partially supported by the relevant literature (63, 64). But by taking empirical measurements and/or conducting meta-analyses using different measurement scales, samples, databases, and taking data from different countries, religious beliefs, and nations, most related studies had found that religious beliefs and participation in religious behaviors could improve happiness, subjective well-being, and mental health (65–73). For example, Cohen-Zada and Sander (68) used repeal as an instrumental variable for church attendance and provided direct evidence that church attendance had a significant positive effect on happiness; the study of Kortt et al. (66) provided strong evidence of an association between attendance at religious services and life satisfaction in the Australian social context; Van Cappellen et al. (71) found that religious beliefs could increase happiness through positive emotions of self-transcendence. In this context, the primary mechanisms of religious belief at play were more social support from believers and more optimism toward evil deeds (65–67). As Zou et al. (74) pointed out, the positive aspects of religion could help people accept failure and negative experiences as a part of life and make peace with them. When like-minded people come together through religion, they may share similar faith and beliefs forming a strong social core for supporting one another. This may not only help prevent depression, but also help in the recovery of depressed persons. Considering the above differences, this study further analyzed the relationship between the two and found that 85% of the religious believers in this study's sample came from lower social classes such as peasants and self-employed workers, and they also had relatively low levels of education. These factors might contribute to the inherently high levels of depression among the religious group in China. Even with the help of their religious beliefs, their depression levels were significantly higher than those without religious beliefs. At the same time, religious organizations in China received less encouragement and support from the government (75, 76), and disclosure of religious beliefs could be a stressor that affected one's mental health (77), which might explain the higher levels of depression among religious believers in this study.

In addition to religious factors, the influence of other control variables on group depression levels is further explored. Firstly, physical activity and exercise can improve depression, and regular exercise can reduce the risk of depression (78). Improving physical health may also contribute to improving mental health, including fewer symptoms of anxiety and depression (79). Therefore, people who exercise regularly and have higher levels of physical fitness, have lower levels of depression. Second, the amount of social capital an individual has also affects his or her level of depression. Social capital includes social trust and social relations (80). People who believe that society is fair, tend to have a stronger sense of social trust, and people with a high degree of social interaction tend to have more social connections. That is, these two groups have more social capital, which helps reduce their levels of depression. Finally, strong supportive social relationships can reduce stress and depression (81). Compared with unmarried people, those who are married have strong supportive social relationships with their spouses and children; therefore, their depression levels are relatively low.

## Conclusion

Becoming a state functionary is a major career choice. The degree of depression of state functionaries not only affects their individual quality of life but also relates to the level of public services they provide. The main conclusions from the results are as follows. (1) The full-sample regression analysis demonstrated that depression scores of state functionaries were 1.010 points lower than those of non-state functionaries, and the results were significant at the 1% level. (2) It was found that the degree of depression of state functionaries was lower than that of workers in all other occupational types. (3) Through the heterogeneity analysis, it was found that age, region, and gender affected the depression levels of state functionaries. Specifically, the degree of depression of older state functionaries was lower than that of younger state functionaries, the degree of depression of state functionaries in the three major urban agglomerations was higher than that of those in the other provinces, and female state functionaries had a lower degree of depression than male state functionaries.

On the basis of the above conclusions, we put forward the following proposals. First, compared to state functionaries, non-state functionaries have higher levels of depression. The reasons for this are poorer pay and benefits. To improve the mental state of non-state functionaries, there is a need to increase the minimum income standards and job security levels for the general workforce so that their risk of mental health problems due to job insecurity, low income, and lack of rest is reduced. Second, it is important to pay attention to the mental health of young state functionaries, considering that younger state functionaries have worse mental health. Third, considering that female state functionaries are in a better state of mental health than male state functionaries, it is necessary to reasonably guide women to join the state functionaries while paying attention to the mental health of men in the state functionaries. Fourth, considering the poor psychological condition of state functionaries in economically developed regions, we need to provide additional subsidies and strengthen psychological counselling for state functionaries in economically developed regions to alleviate their psychological problems caused by their relatively low incomes, stressful lives and high intensity of service.

## Limitations

This study has three limitations: (1) with regard to sample selection bias, this study controlled only for the observable selection bias and not for the unobservable selection bias; (2) due to the limited data, this study did not explore the time effect of serving as a state functionary on depression; and (3) this study did not analyse the specific mechanisms that cause the different depression levels among state functionaries.

## Data Availability Statement

The data used in this study was obtained by applying to the Center for Social Survey of Sun Yat-sen University [cssdata@mail.sysu.edu.cn]. Please contact the corresponding author if necessary. Email: home@zuel.edu.cn.

## Author Contributions

LH and HY: conceptualization. KW: method, software, and visualization. KW, ZZ, and JW: validation. JW: formal analysis and investigation. YWa: resources. HY: data curation and project administration. ZZ, JW, and TL: writing—original draft preparation. LH, LY, YWu, ShZ, and SiZ: writing—review and editing. LH: supervision and funding acquisition. All authors have read and agreed to the published version of the manuscript.

## Funding

This research was funded by the Fundamental Research Funds for the Central Universities (Program No. 2722021EK008) and the Humanities and Social Sciences Fund of the Ministry of Education (Grant Number: 19YJC790167).

## Conflict of Interest

The authors declare that the research was conducted in the absence of any commercial or financial relationships that could be construed as a potential conflict of interest.

## Publisher's Note

All claims expressed in this article are solely those of the authors and do not necessarily represent those of their affiliated organizations, or those of the publisher, the editors and the reviewers. Any product that may be evaluated in this article, or claim that may be made by its manufacturer, is not guaranteed or endorsed by the publisher.
